# Going beyond Cellulose and Chitosan: Synthetic Biodegradable Membranes for Drinking Water, Wastewater, and Oil–Water Remediation

**DOI:** 10.1021/acsomega.3c01699

**Published:** 2023-07-03

**Authors:** Ria Sen Gupta, Paresh Kumar Samantaray, Suryasarathi Bose

**Affiliations:** † Department of Materials Engineering, Indian Institute of Science, Bangalore, Karnataka560012, India; ⊥ International Institute for Nanocomposites Manufacturing (IINM), WMG, University of Warwick, Coventry CV4 7AL, U.K.

## Abstract

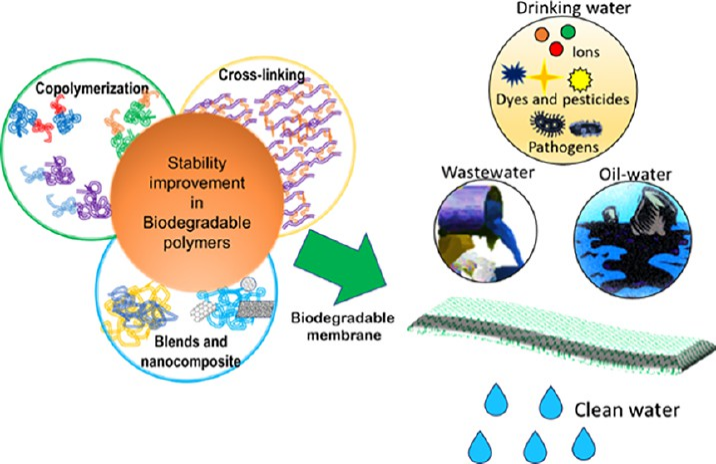

Membrane technology is an efficient way to purify water, but it generates non-biodegradable biohazardous waste. This waste ends up in landfills, incinerators, or microplastics, threatening the environment. To address this, research is being conducted to develop compostable alternatives that are sustainable and ecofriendly. Bioplastics, which are expected to capture 40% of the market share by 2030, represent one such alternative. This review examines the feasibility of using synthetic biodegradable materials beyond cellulose and chitosan for water treatment, considering cost, carbon footprint, and stability in mechanical, thermal, and chemical environments. Although biodegradable membranes have the potential to close the recycling loop, challenges such as brittleness and water stability limit their use in membrane applications. The review suggests approaches to tackle these issues and highlights recent advances in the field of biodegradable membranes for water purification. The end-of-life perspective of these materials is also discussed, as their recyclability and compostability are critical factors in reducing the environmental impact of membrane technology. This review underscores the need to develop sustainable alternatives to conventional membrane materials and suggests that biodegradable membranes have great potential to address this challenge.

## 1 Introduction

Water forms the basic foundation for fostering and functioning life on Earth. Although access to safe drinking water is a fundamental right, water scarcity issues are a global concern today, primarily due to rapid industrial, agricultural, and technological growth triggered by the growing population. This has led to overuse and contamination of existing freshwater sources.^1,2^ A report by the World Health Organization discussed that waterborne diseases like diarrhea could be curbed by 35% if basic water hygiene and sanitation are in place.^3,4^ Lack of proper sanitation may also lead to emerging pathogens such as the Ebola virus and the recent SARS-CoV-2 entering the water system, posing implications for human health.^5,6^ According to another report, it is estimated that two-thirds of the world’s population will be living in water-stressed regions by 2050, with continuous or recurring freshwater shortages.^7,8^ Therefore, low engineering costs and energy-efficient strategies to purify existing water resources are desirable and critically essential.

Membrane separation with a tailored pore size offers strategic elimination of a wide range of contaminants, including particulate matter, colloids, persistent recalcitrants, waterborne pathogens like bacteria, fungi, algae, protozoa, and viruses, and even ions and heavy metals. Different technological interventions have enabled scientific progress in desalination, heavy metal removal, pathogen removal, and electrodialysis, among others .^9−13^ Different membrane processes like reverse osmosis (RO), nanofiltration (NF), ultrafiltration (UF), and microfiltration (MF) have been deployed in the past decade with some of the successful polymer materials like cellulose acetate (CA), polysulfone (PSU), polyvinylidene fluoride (PVDF), polydimethylsiloxane (PDMS), polyvinyl chloride (PVC), polyethylene (PE), polypropylene (PP), polyethersulfone (PESU), polyamide (PA), polyacrylonitrile (PAN), and poly(vinyl alcohol) (PVA).^3,14^ Further, with engineered strategies incorporating biocidal and antifouling agents, such membranes’ shelf lives have also been enhanced.^1^ The key concern lies in the prolonged usage and disposal consideration after the intended end-use of these membranes.

Most of the commercial membranes are nonbiodegradable. Safe disposal after the end-use of these membranes should be considered, which is often overlooked. These wastes may end up like other plastic in landfills or get incinerated, resulting in increased carbon dioxide (CO_2_) emissions and contributing to global warming.^15^ Further, microplastic pollution from generally discarded plastics is a growing concern. Although the potential toxicological effects associated with microplastics are mostly unknown, at a considerably high concentration, they can negatively impact organisms in aquatic environments.^16^ Faure et al. analyzed the microplastics in Lake Geneva and the Mediterranean Sea, and membrane microplastics < 2 mm were also observed in their analysis of samples.^17^ Over the due course of their continued long-term usage, chemical cleaning agents like acids, oxidants, surfactants, bases, and chelating agents used to reduce fouling can trigger polymer membrane aging and release microplastics in water treatment plants.^18,19^ Among different membrane materials, PESU, PVC, and PP microplastics have also been detected in drinking water and wastewater treatment processes.^19^ While it is critical to study this aspect of membrane science in due course, a way forward could be envisaged using environmentally friendly, biodegradable plastics.

Biodegradable bioplastics have gained quite a lot of attention due to their innate ability to degrade under controlled natural and composting processes.^20,21^ In particular, biodegradable polymers can potentially break down into natural byproducts such as water, carbon dioxide and/or methane, and biomass. As a result, they contribute significantly less to environmental pollution compared to their commercially manufactured counterparts. The latest report of European Bioplastics from nova-Institute in 2019 estimates the current production of bioplastics at 2.11 million tonnes and projects that it will increase up to 2.42 million tonnes by 2024.^22^ Biodegradable polymers such as poly(lactic acid) (PLA), poly(caprolactone) (PCL), poly(butylene succinate) (PBS), poly(vinyl alcohol) (PVA), and poly(hydroxyalkanoate) (PHA) have been slowly realized for sustainable membrane separation applications. PHA can be derived from microbial biosynthesis, while some biopolymers such as starch, cellulose, lignin, and chitosan are derived directly from biomass. PLA and poly(glycolic acid) (PGA) can be derived from biobased precursors, while PVA and PCL are synthesized from petrochemical derivatives.^23^ Despite their diverse origins, the potential capabilities of these materials to assimilate into environmentally accepted substances make them potential candidates for sustainable remediation.

In contrast to the cellulosic and chitosan derivatives, newer biodegradable bioplastics like biodegradable biopolyester exhibit better thermomechanical properties, are food-contact-compliant, and have fair to good acceptability to various chemical environments. These polymers and their composites can be potential materials to envisage sustainable water purification with a low environmental impact. The life-cycle analyses of bioplastics as per European Bioplastics also indicate that they can also reduce the emissions of CO_2_ by 30–70% compared to conventional plastics.^24^ While the current production of PHA, PLA, and starch blends, among others, accounts for ∼60% of global bioplastics (over 1.2 million tonnes), the production is expected to increase to 1.8 million in 2025 due to significant growth and investments in PHA and newer investments for PLA in the US and Europe.^25^

This review aims to comment on the feasibility of newer biodegradable bioplastics beyond cellulose and chitosan as alternatives to the existing membrane materials and then highlight the key opportunities for developing sustainable membranes with low plastic waste. To examine this, we compare various key parameters like the price, carbon footprint in primary production, thermomechanical characteristics (like tensile strength, fracture toughness, extension at break glass transition temperature, and melting temperature), flexibility to processing, maximum service temperature, food compliance, durability in fresh and saline water, durability in weak acids and alkali, durability to organic solvents, and UV radiation. The key standpoints that may be adopted to improve the characteristics are also highlighted. Moving forward, we also discuss recent trends and advances made by the scientific community in the design and application of synthetic biopolyester membranes to water remediation applications and finally discuss the scope and prospects of these materials in sustainable environmental remediation applications.

## 2 Feasibility Analysis of Biodegradable Bioplastics in Membrane Applications

**Figure 1:**
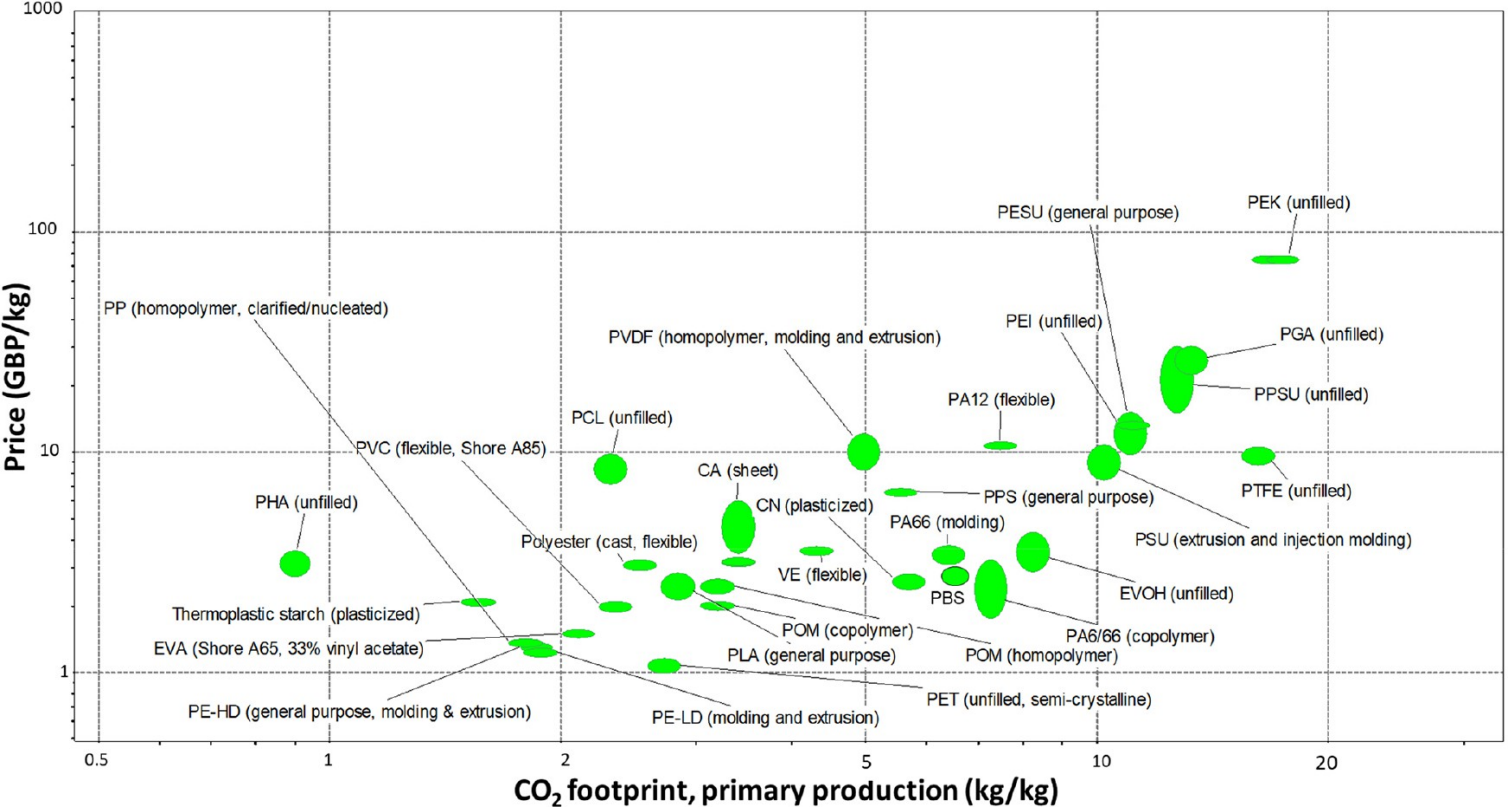
Price vs CO_2_ footprint, primary production (kg/kg), for conventional membranes and biodegradable bioplastics created using CES EduPack 2019.

The first criterion for comparison is the CO_2_ footprint involved during primary production (kg/kg) and the price per kilogram of biodegradable bioplastics compared to the conventional membrane materials. For this, we used CES EduPack 2019 database of polymers. Figure 1 shows a comparison of all of the current membrane materials alongside biodegradable bioplastics. It can be observed that most of the membrane materials like PVDF, CA, PSU, PVC, PESU, PE, and PP have prices ranging from 1 to 10 £/kg, while some materials used in specific purification like polytetrafluoroethylene (PTFE) and polyether ketone (PEK) are toward the costlier end. Available biodegradable polymers like poly(hydoxyalkonate) (PHA), PLA, PCL, and PBS also lie within the same price range. While examining the carbon footprint, except polyglycolic acid (PGA), other biodegradable bioplastics have a primary production footprint of of <5 kg/kg CO_2_. In this case, polyamides, PVDF, and PSU, among others, have carbon footprints higher than those of biodegradable bioplastics.

The next criteria critical in membrane application are the tensile strength and the polymer material’s maximum service temperature. Service temperature helps in understanding the stability of the polymer material up to a certain temperature. This allows users to choose materials for high-temperature membrane processes such as membrane distillation and solar evaporator applications.^26,27^

**Figure 2:**
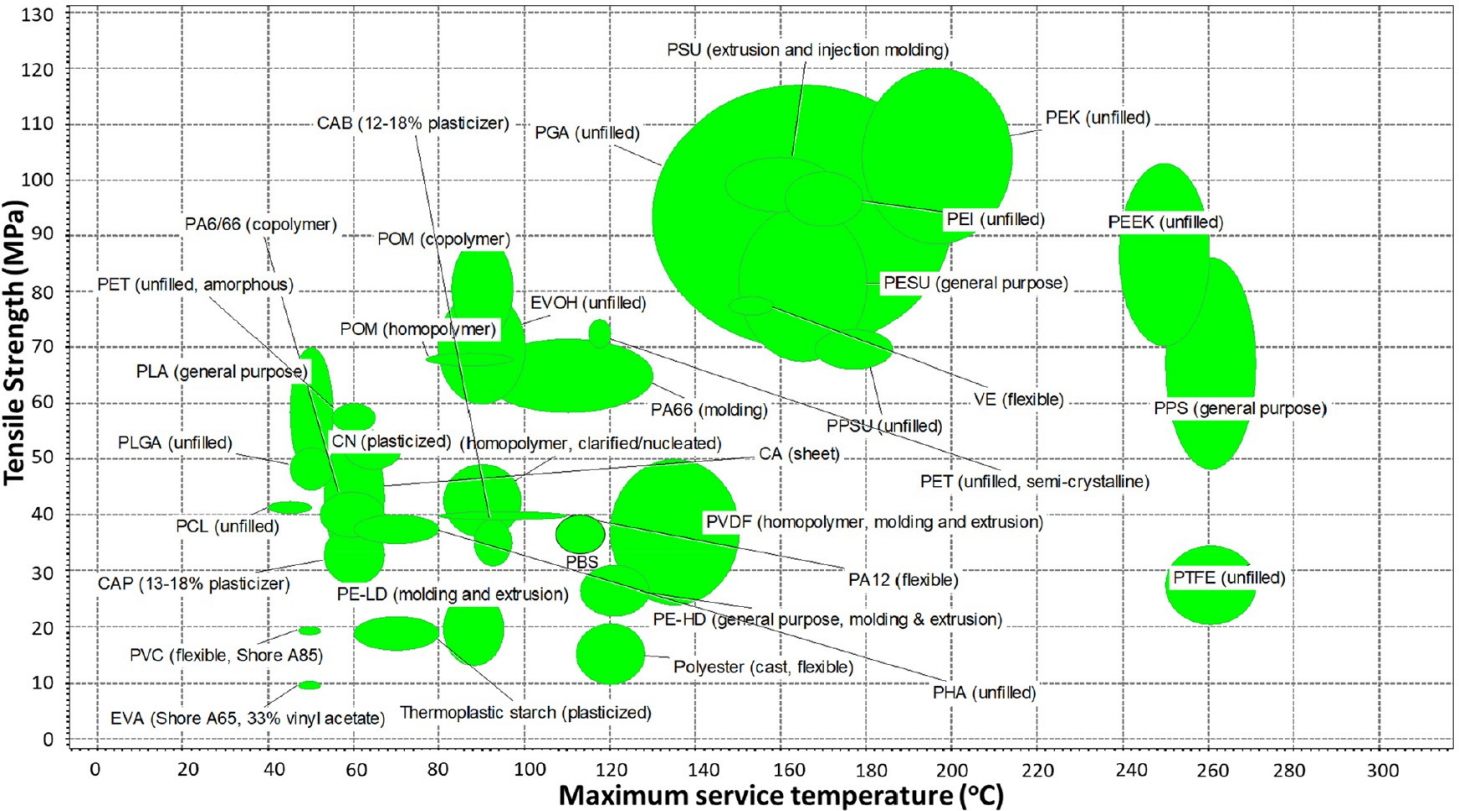
Tensile strength vs maximum service temperature for conventional membranes and biodegradable bioplastics created using CES EduPack 2019.

Figure 2 shows the tensile strength vs maximum service temperature of conventional membrane materials compared with biodegradable bioplastics. While poly(lactic-*co*-glycolic acid) (PLGA), PHA, CA, cellulose nitrate (CN), cellulose acetate butyrate (CAB), and PLA have comparable tensile strengths compared to PVDF, they are limited by the maximum temperature of <100 °C. Among biodegradable bioplastics, PGA has the highest tensile strength and a diverse maximum service temperature range from 140 to 200 °C, which is comparable to those of PSU, PESU, and PEK. Table 1 compares the thermomechanical properties of conventional membrane materials and biodegradable bioplastics. It can be observed that most of the biodegradable bioplastics have tensile properties, including superior elongation at break. Some biodegradable bioplastics such as PGA and PLA are brittle. These materials can be used in tubular, capillary, and hollow fiber modules, like traditional ceramics. For flat sheet configurations, the polymers must be modified by blending or copolymerization. It is also noted that although the polymer tensile strength shown here is that of the pristine material (without pores/voids), membranes derived from such materials can have lower mechanical strength. For example, in nonsolvent-induced phase separation, micro- and macrovoids generated in the membranes as the consequence of solvent and nonsolvent exchange can reduce the tensile strength of the polymer membrane.^28^ Similar observations of microvoid formation also hold when the solvent is allowed to evaporate in the thermally induced phase separation process. However, it is possible to tailor and control the mechanical properties of the membranes by increasing the coagulation bath solvent concentration that helps in the formation of the spherulitic morphology, which improves mechanical properties; the increase in the bath temperature induces the formation of a bicontinuous morphology free of macrovoids. Jung et al. improved the mechanical properties of their PVDF membranes from 0.9 to 6.1 MPa using these principles.^28^ In microporous membranes derived from a combination of melt and cold stretching of semicrystalline polymers, annealing and stress-induced crystallization play critical roles in the mechanical properties of the final membranes. It is well-known that increasing the crystallinity of a polymer enhances its tensile strength. Sadeghi et al. demonstrated that annealing PP films improved the crystal phase orientation and had a weaker effect on the amorphous phase.^29^ The annealing time, the temperature set for annealing, and the stress applied during annealing also play critical roles in the mechanical properties of the final film. Annealing helps the removal of defects in the crystalline structure, enables the thickening of lamellae, and improves the orientation and in turn the mechanical properties.^30^

**Table 1:**
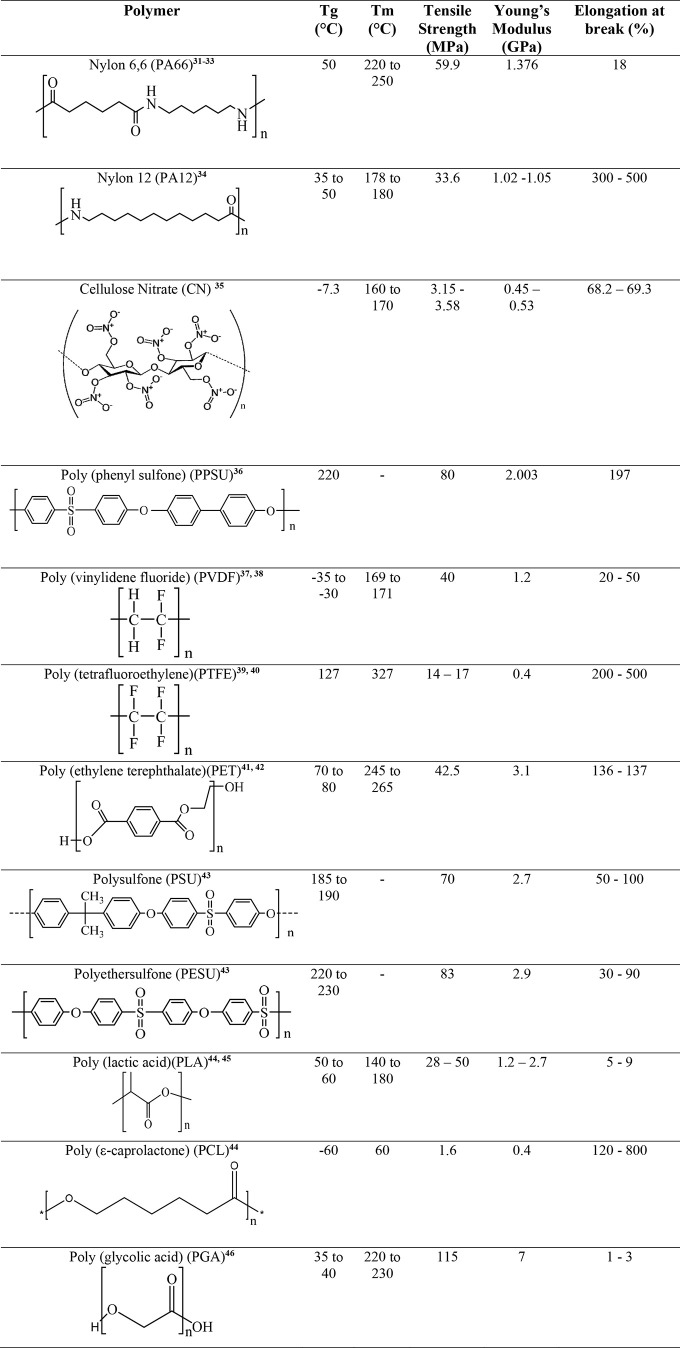

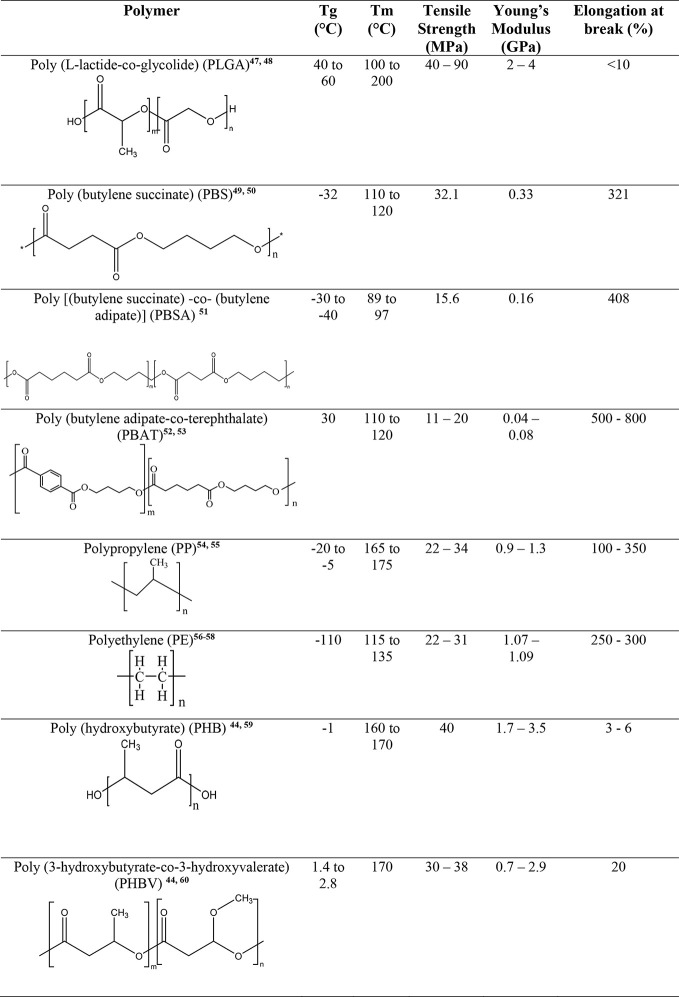
Thermomechanical Properties^a^ of Conventional Membrane Materials and Biodegradable Bioplastics (31,32,33,34,35,36,37,38,39,40,41,42,43,44,45,46,47,48,49,50,51,52,53,54,55,56,57,58,59,60)

a The shown thermomechanical properties are from the literature references and are dependent on the material grade, crystallinity, and monomer ratios. Further, mechanical properties change as a function of the cross-head speed, so values should not be considered absolute.

**Figure 3:**
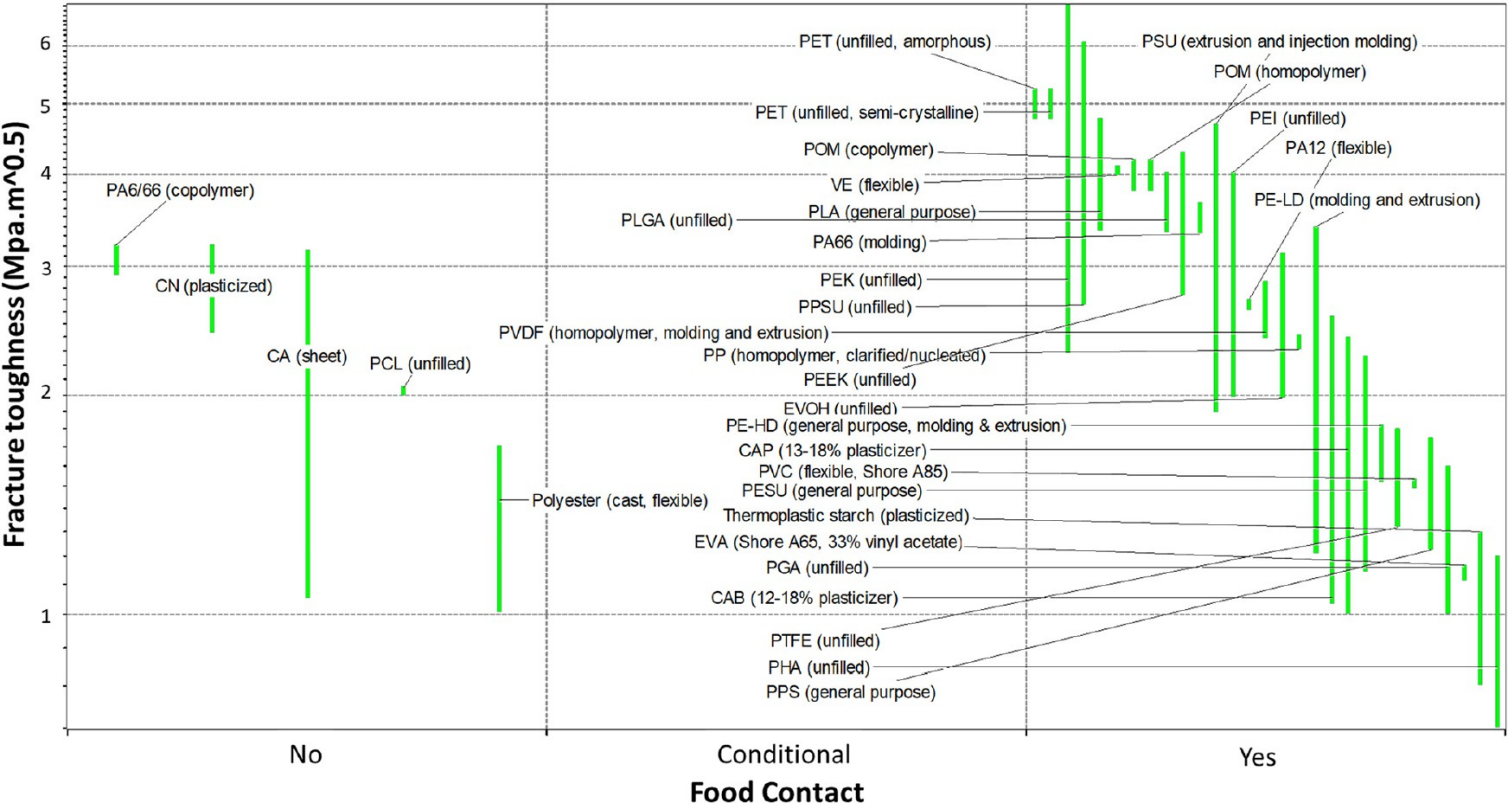
Fracture toughness vs food contact compliance for conventional membranes and biodegradable bioplastics created using CES EduPack 2019.

Apart from maximum tensile strength, fracture toughness also plays a key role in membrane separation (especially while preparing mixed-matrix membranes).^61^ In this regard, PLGA and PLA have higher fracture toughness compared with PVDF, PESU, PVC, PE, polyesters, PPS, PTFE, and polyamides. Except for CA, CN, PCL, and PA6/66 polyamide, all the other grades are food-contact-compliant, refer to Figure 3. It should be noted here that while most of the newer biodegradable bioplastics are food-contact-safe, some of the cellulosic derivatives are not suitable for such applications. This might limit their usage in beverage purification processes.

**Figure 4:**
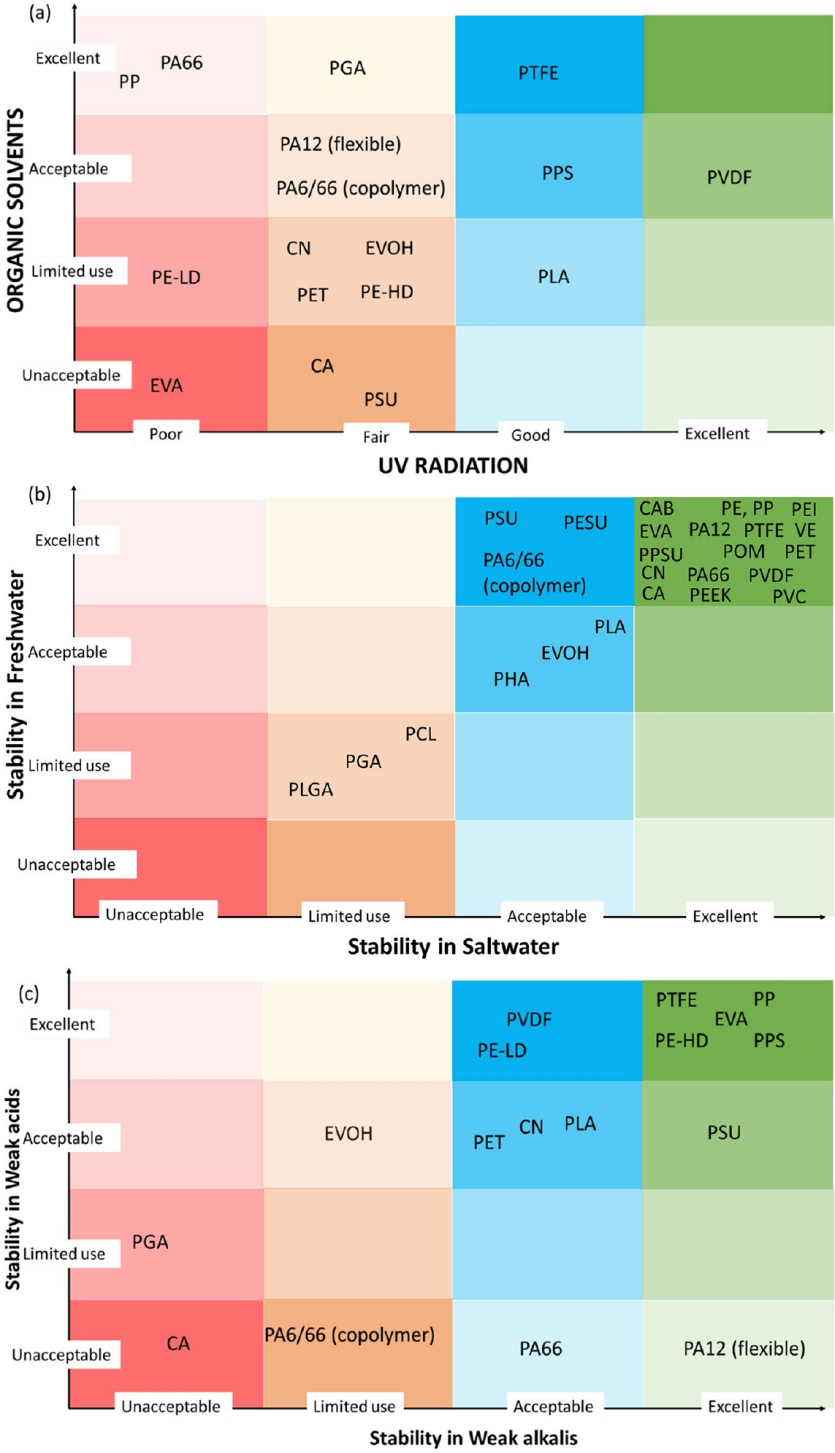
Stability to (a) organic solvents vs UV radiation, (b) freshwater vs saltwater, and (c) weak acids vs weak alkalis. Along the *y*-axis, the darker red and orange shades indicate unacceptable or limited use, while the lighter colors of the same shade move toward acceptable and excellent considerations. Conversely, the lighter versions indicate unacceptable or limited use for blue and green shades, while the darker versions indicate acceptable or excellent considerations. Along the *x*-axis, colors from red to green indicate unacceptable to excellent considerations, respectively. Data extracted from CES EduPack 2019 and replotted.

Water filtration membranes not only encompass domestic water purification but also involve wastewater remediation as well. Wastewater contains organic waste, pathogens, heavy metals, pesticides, dyes, organic solvents, etc. One of the key membrane techniques to purify wastewater is using photocatalytic membranes.^62,63^ For desalination, the membranes should show relative stability to a highly saline environment. Hence, it is important to evaluate the stability of these polymer materials in organic solvents, UV radiation, freshwater, saltwater, weak acids, and weak alkalis. Figure 4(a–c) shows this comparative assessment using CES EduPack 2019. It can be seen that all of the biodegradable polymers and conventional polymers like CN, PET, CA, and CN have limited use in organic solvents. Most of the biodegradable materials have acceptable stability to weak acids and alkalis, with exceptions to PGA. Among biodegradable polymers, PLA is stable to UV, while PGA is fairly stable to UV irradiation. It can also be seen that virgin PLGA, PGA, and PCL have limited use in saltwater as well as freshwater because they hydrolyze easily in the presence of water. This is a key challenge in using these polymers in water filtration membranes without modifications. Suggested engineering strategies to improvise this drawback are discussed in section 3, while the potential applications of these new biodegradable bioplastics are summarized in Table 2. It is to be noted that with the functional modifications these membranes can used in drinking water, wastewater, and oil–water separation only. Desalination capabilities of these materials after chemical modifications are not clearly understood in the literature and require additional research.

**Table 2:** Potential Membrane Separation Applications of Emerging Biodegradable Bioplastics and Their Composites

biodegradable bioplastic and its composites	potential separation applications
poly(butylene succinate) (PBS)	retention of *Lactobacillus plantarum* cell suspensions^64^
	removal of total dissolved solids, chemical oxygen demand, and turbidity^65−67^
	dye adsorption^68^
poly(ε-caprolactone) (PCL)	adsorption of peanut oil, motor oil, and diesel oil and separation of oil–water emulsions^69−73^
	antibacterial water filtration^74^
	removal of anionic pollutants and heavy metals such as nitrates, sulfates, Pb, Cd, and Zn^75,76^
	removal of dye contaminants such as Congo Red^77^
poly(lactic acid) (PLA)	oil–water separation^78−80^
	recyclable dye adsorption^78^
	separation of methanol/methyl *tert*-butyl ether azeotropic mixture^81^
	bacteria-resistant membrane surface with self-cleaning abilities^82^
	antifouling membranes^83^
	retention of *Lactobacillus plantarum* cell suspensions^84^
	membranes with high clearance of urea and lysozyme^85^
poly(hydroxyalkanoate)	highly efficient bacteria filtration^86−88^
poly(vinyl alcohol)	high rejection of various latex nanoparticles^89^
	oil–water separation^90−92^
	leachate treatment^93^ and desalination^93,94^
	dye removal^95−98^
	adsorption of heavy metal ions such as Cu^2+^ and Pb^2+^^99^
	isopropanol dehydration^100^

## 3 Improving the Properties of Biodegradable Polymers

### 3.1 Polymer and Nanoparticle Blending

Blending biodegradable polymers with nonbiodegradable membrane materials like PE, PP, PPSU, PVDF, PET, PA, and PAN can impart improved mechanical strength, good water and thermal stability, and tolerance to harsh chemicals, which can potentially deteriorate the membrane performance. The biodegradable and nonbiodegradable polymers may not be chemically compatible and might need a small amount of a reactive chemical agent termed as a compatibilizer to stabilize both the phases and yield synergistic properties mutually. Using compatibilized blends, green composites can be tailored to have a good elongation at break. Further, using blending, the dependence on the existing nonbiodegradable polymer materials will be reduced while not completely replacing them from the consumption chain. Yang et al. blended PP with PGA using maleated ethylene octene copolymer and attapulgite as a reactive compatibilizer, which enhanced the toughness, extension at break, tensile strength, and thermal stability of the system.^101^ Nuñez et al. blended PLA with PP using maleic anhydride and a clay-based compatibilizer. While PLA is inherently brittle, the blend has improved thermal and mechanical stability.^102^ Aseri et al. blended PVDF with PLA to fabricate hollow fiber membranes. Incorporation of <1 wt % PLA significantly improved the membrane flux per bar from 30 to 376.7 L/m^2^·h while maintaining a 95–97% rejection of humic acid. PLA also improved the antifouling nature of PVDF membranes by improvising the flux retention after bovine serum albumin fouling.^103^ Li et al. blended poly(l-lactic acid) (PLLA) with PVDF using a reactive compatibilizer containing a glycidyl methacrylate derivative at a composition of 70/30/20 w/w, which improved the mechanical properties of the blend while maintaining optical clarity.^104^ Yang et al. designed a poly(styrene-*co*-(glycidyl methacrylate)-*co*-(maleic anhydride))-based compatibilizer for stabilizing polyamide 11 (PA 11) and PLLA blends. With just a 3% addition of this compatibilizer, the elongation at break and tensile strength were enhanced to 411% and 57.9 MPa, respectively.^105^ Biodegradable polymers can themselves be blended with each other to yield compostable blends with good mechanical properties. In such cases, the extension at break, tensile strength, and compostability are dominated by the major polymer phase. For example, PBS is suitable for industrial composting and blends with PBS as the major phase (>60%) will be only compostable under industrial composting conditions, while the strain at break will be dependent on the concentration used.

Apart from conventional membrane materials, nanofillers can also be melt blended or solution mixed with the biodegradable polymers and their blends. Nanofillers can be inorganic, carbonaceous fillers, clays, and other organic nanofillers. Inorganic nanofillers are metals and their metal oxides, such as silver, copper, zinc oxide, copper oxide, and titanium dioxide, to name a few. The major role of the inorganic nanofillers is to provide antimicrobial performance to the membrane.^14,106^ However, inorganic nanofillers like titanium dioxide can impart antimicrobial properties to the overall composite as well as reduce the water vapor permeability of the composite.^107^ Among carbonaceous fillers, carbon nanotubes, graphene, and graphene oxide have been commonly used as conventional membrane materials for water treatment processes. Such fillers and their modifications can also be used with biodegradable polymers, as well. For example, Kim et al. blended PLA with graphene oxide and carbon nanotubes as hybrid co-filler. With the 0.4 wt % addition of this hybrid filler, the tensile strength of the composite film increased by 75% and the Young’s modulus increased by 130%.^108^

Inclusion of clay can delay the hydrolytic degradation of biodegradable polymers like PLA. They can cause a delay in polyester degradation due to the barrier effect and lower the available surface for hydrolysis.^109^ Chen et al. melt blended organic montmorillonite with poly(l-lactic acid) (PLLA). With the addition of 1 wt % organic montmorillonite via melt processing, followed by sample annealing at 80 °C for 30 min, the hydrolytic degradation rate of PLLA was only ∼0.25% with a residual polymer weight of >95%.^110^ This was because hydrolytic degradation occurs in the amorphous domains of the polymer chain. Annealing enabled the crystallization of PLLA in the presence of the nanoclay. Fukushima et al. also observed a delayed degradation of the PLA matrix in the presence of sepiolite.^111^ Zhou et al. blended 5 wt % cationic montmorillonite and anionic hydrotalcite clay with PLA. The thermal stability was improved, and the hydrolytic degradation rate constants of both composites were lower than that of pristine PLA. In particular, the anionic nanocomposite neutralized the catalytic effect of the carboxylic group of PLA, enabling enhanced water stability.^112^ In organic nanofillers, chitosan, nanocrystalline cellulose, nanofibrillated cellulose, and bacterial nanocellulose are most commonly used with biodegradable polymers to improve the membrane performance details, which are discussed in section 4.

### 3.2 Copolymerization and Cross-Linking Approaches

The incorporation of long chains of aliphatic groups increases the flexibility of the copolymer and decreases the glass transition temperature. Incorporating rigid pendant units like benzene rings can increase the mechanical strength and the glass transition temperature.^23^ For example, the crystallinity and melting temperature of PBS could be lowered using adipic acid as a copolymerization subunit to form PBSA. This also results in a faster biodegradation rate for PBSA compared to homopolymer PBS.^23^

Conversely, using water-insoluble monomers, such as aliphatic acrylate derivatives, can improve the water stability of the final copolymer. Elisseff et al. designed poly(l-lactic acid-*co*-l-aspartic acid) by the ring-opening polymerization of poly(lactic acid-*co*-lysine) in the presence of aspartic acid and UV polymerized the same in the presence of hydroxyethyl methacrylate, which resulted in a flexible yet water-insoluble copolymer gel.^113^ Kaczmarek et al. photopolymerized PLA in the presence of polyacrylates in an equal ratio, which resulted in cross-linked gels that were insoluble.^114^ Zhu et al. fabricated a block copolymer of PLA with hydroxyethyl methacrylate and blended it with PLA to form membranes. With just 15 wt % PLA–poly(hydroxyethyl methacrylate) copolymer, the flux was ∼236 L/m^2^·h with a flux retention close to 80% after being subjected to protein foulant bovine serum albumin.^115^ Yu et al. improvised the mechanical, thermal, and water stability of PLA by fabricating polysulfone-grafted PLA. The samples had an elongation at break of 60%, a tensile strength of 3.34 MPa, and a bovine serum albumin rejection of 95%.^85^ It is to be noted that the choice of monomers for copolymerization is made depending on the type of membrane separation application. If the membranes require high flexibility and durability, aliphatic monomer units, which can form long chains of the −CH_2_ network, can be selected. If antifouling and antibacterial response are both desired, monomers with antimicrobial subunits like quaternary ammonium, sulphonium, phosphonium, and amines^14^ can be copolymerized with the biodegradable polymers.

Chemical cross-linking reactions also improve the water stability and rate of hydrolysis of biodegradable polymers. For instance, Xiong et al. synthesized a copolymer of NVP (1-vinyl-2-pyrrolidone) and VTES (vinyltrimethylsilane), P(VP-VTES), to cross-link PLA chemically. It was observed that the surface cross-linking increased the crystallinity of PLA by up to 38%, and the sample withstood 100 °C of hydrothermal treatment with no depreciation in filtration efficiency. The dry membrane flux recovery was ∼98%.^116^ Xue et al. used bis(*tert*-butyl dioxy isopropyl) benzene to cross-link PLA/PBAT-based blend blown films. While the mechanical properties were retained, the enzymatic degradation of 20:80 PLA/PBAT was significantly retarded to <0.25 % with just 0.1% of the cross-linking agent.^117^ Rhim et al. used sulfosuccinic acid (5–30 wt %) to cross-link PVA. It was observed that the membranes only became swollen under boiling water even after remaining in the same condition for a week due to cross-linking by SSA.^118^ Yang used glutaraldehyde (GA) as a cross-linking agent for PVA-TiO_2_ to enhance the chemical, thermal, and mechanical stabilities.^119^

## 4 Recent Progress of Biodegradable Membranes toward Water Purification

### 4.1 Poly(butylene succinate) (PBS) Based Membranes

PBS is a biodegradable aliphatic polyester. It exhibits excellent thermoplastic processability with high crystallinity and a *T*_g_ below room temperature.^120^

This polyester is considered a biomass plastic since its monomers, i.e., succinic acid and 1,4-butanediol, can be produced from biomass. While succinic acid can be produced via bacterial fermentation and chemical routes, 1,4-butanediol can be generated from its acid counterpart via hydrogenation.^121^ PBS possesses several desirable properties, such as high availability, good processability, suitable flexibility, excellent impact strength, and high chemical resistance. These have turned it into one of the most sought-after biodegradable polymers.^122,123^ Besides, PBS can also be processed at much lower costs as compared to PCL and PLA.^124^

Tanaka et al. reported the synthesis of microporous membranes via nonsolvent and thermally-induced phase separations using chloroform solutions.^64^ Studies revealed that the polymer concentration during membrane preparation significantly affected its permeation and retention properties. Nearly 99% retention of *Lactobacillus plantarum* cell suspensions were reported. However, inadequate mechanical properties such as the low tensile strength and relative hydrophobicity of PBS limited the prepared membrane’s commercial application.

With the vision of increasing the mechanical strength of PBS, Ghaffarian et al. developed blend membranes of CA and PBS, thus creating biodegradable membranes of CA/PBS with varying PBS concentrations. With 20 wt % CA, total turbidity removal was observed along with the removal of nearly 80% of total dissolved solids from wastewater.^67^

In a different work by the same group, a PESU and PBS-based blend was used to improve the mechanical, thermal, and film-forming characteristics of PBS. With just 15% PESU, 75% of chemical oxygen demand, 100% turbidity, and 40% of total dissolved solids were removed from the wastewater.^66^

In another work, Ghaffarian et al. modified PESU/PBS blend membranes with a PEG additive, which was found to increase the hydrophilicity of the membranes. Nearly 100% turbidity, 60% chemical oxygen demand, and 30% total dissolved solids were removed by PEG400-modified membranes with a PEG concentration of 15%.^65^

Bahremand et al. modified CA/PBS blend membranes with a hydrophilic additive, i.e., Dextran (DEX). The additive enhanced the biodegradability, hydrophilicity, permeation flux, and antifouling properties of the blended membrane. The fluxes for pure water and wastewater were measured at a transmembrane pressure of 3 bar and increased from 27.93 to 70.90 LMH and from 10 to 18 LMH, respectively, when the additive concentration was increased to 2 wt %.^125^

**Figure 5:**
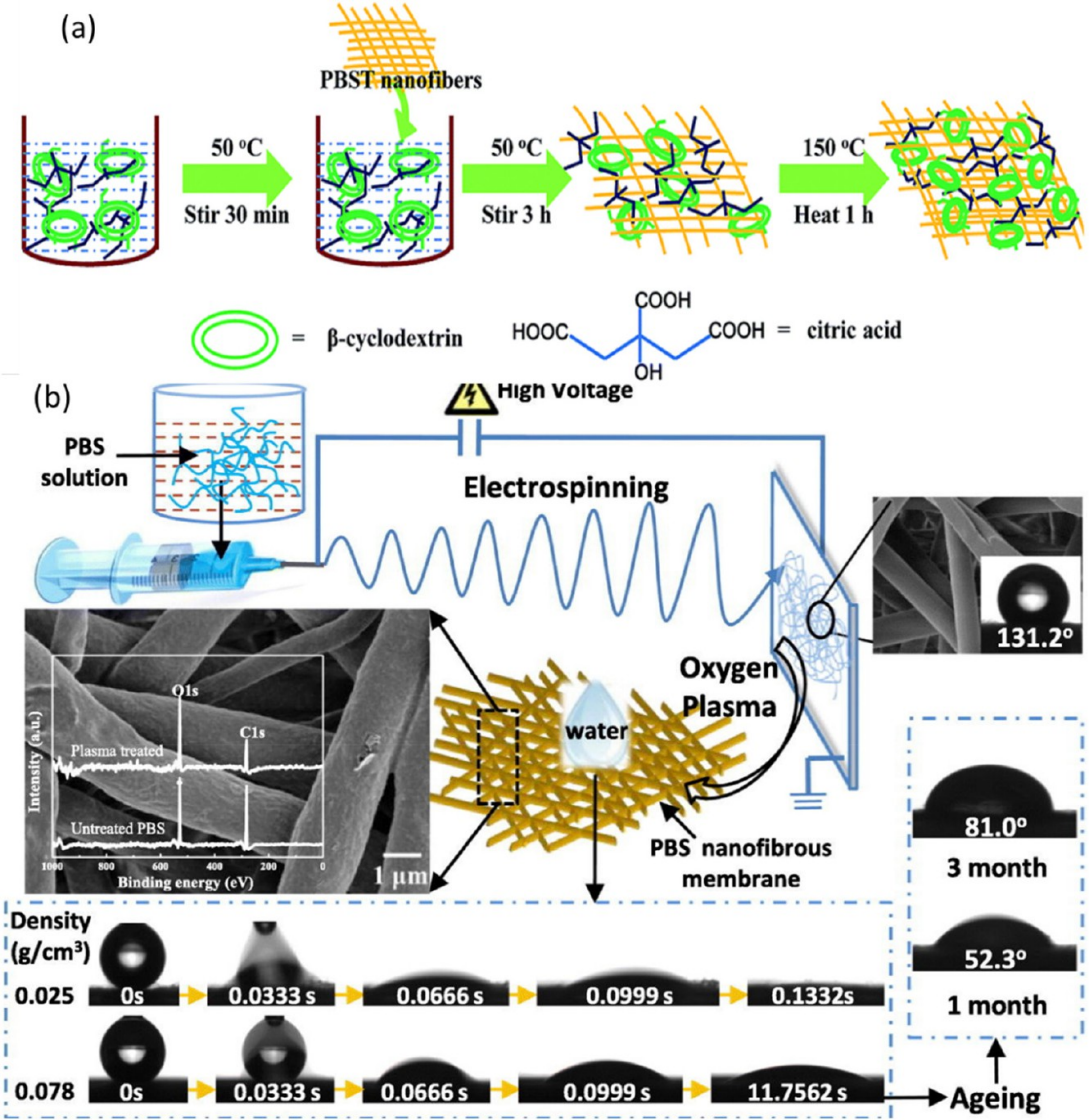
(a) Schematic for the fabrication of PBST/CDP nanofibrous membranes. Reproduced with permission from ref (68). Copyright 2016 Royal Society of Chemistry. (b) Biodegradable poly(butylene succinate) nanofibrous membrane treated with oxygen plasma for super hydrophilicity. Reproduced with permission from ref (126). Copyright 2020 Elsevier.

Wei et al. fabricated biodegradable poly(butylene succinate-*co*-terephthalate) (PBST) nanofibrous membranes via electrospinning, following which they functionalized the membranes with β-cyclodextrin polymer (CDP) via an in situ polymerization technique.^68^ The as-prepared PBST/CDP nanofibrous membranes exhibited an exceptional adsorption capacity of about 90.9 mg/g toward a methylene blue solution. Refer to Figure 5(a) for schematics.

Gu et al. designed a biodegradable PBS nanofibrous membrane via an electrospinning technique and treated the prepared membrane with low-temperature oxygen plasma to render the membrane with complete hydrophilic properties. The oxygen plasma treatment enhanced the surface roughness and introduced oxygen-containing groups on the surface. These polar functional groups changed the membrane from being original hydrophobic to super hydrophilic. The time required for the water droplets to completely spread out on the surface was less than 0.5 s.^126^ Refer to Figure 5(b) for the schematics.

Ebrahimpour et al. described the preparation of a novel PBS/Al_2_O_3_ nanoparticle composite membrane using the phase inversion method.^127^ The experimental results indicated substantial improvement in surface and mechanical properties for PBS membranes cast in an isopropanol coagulation bath with an increasing Al_2_O_3_ nanoparticle concentration up to 1 wt %. The membranes were compacted at a pressure of 7 bar and exhibited a pure water permeability of 13 L/m^2^·h·bar, 85 L/m^2^·h·bar permeate flux of tomato canning wastewater, a COD rejection of 85%, a TDS rejection of 70%, and a turbidity rejection of 98%.

### 4.2 Poly(ε-caprolactone) (PCL) Based Membranes

PCL is a commercially available and well-known semicrystalline biodegradable polyester. It is prepared by ring-opening polymerization of ε-caprolactone and can be degraded via micro-organisms or by the hydrolysis of its ester linkages.^128,129^ Its superior blend compatibility has been instrumental in its potential applications in biomedicine, packaging, sewage treatment, and the food industry, among others, and is exceptionally safe for human health.^130,131^ PCL has a glass transition temperature of −60 °C along with a melting point of 59–64 °C. It is soluble in a wide range of solvents, including chloroform, dichloromethane, carbon tetrachloride, benzene, toluene, cyclohexanone, and 2-nitropropane, at room temperature. Hence, it can be easily processed using various fabrication techniques. Owing to advantageous properties such as biocompatibility, biodegradability, cost-effectiveness, high mechanical and thermal stability, UV and chemical resistance, and low water absorption, PCL has emerged as a promising material for the fabrication of water remediation membranes.^132^

Yin et al. designed a novel superhydrophobic PCL/PS (polystyrene) composite nanofibrous membrane through solution blow spinning with an airbrush for potential oil adsorption applications.^69^ The saturation adsorption capacities of the composite membrane toward peanut oil, motor oil, and diesel oil were 16.89 20.33, and 12.17 g/g, respectively. After six cycles of reutilization, the adsorption capacities to peanut oil, motor oil, and diesel oil remained at 6.16, 7.46, and 5.33 g/g, respectively, higher than those of the pure PCL membrane.

**Figure 6:**
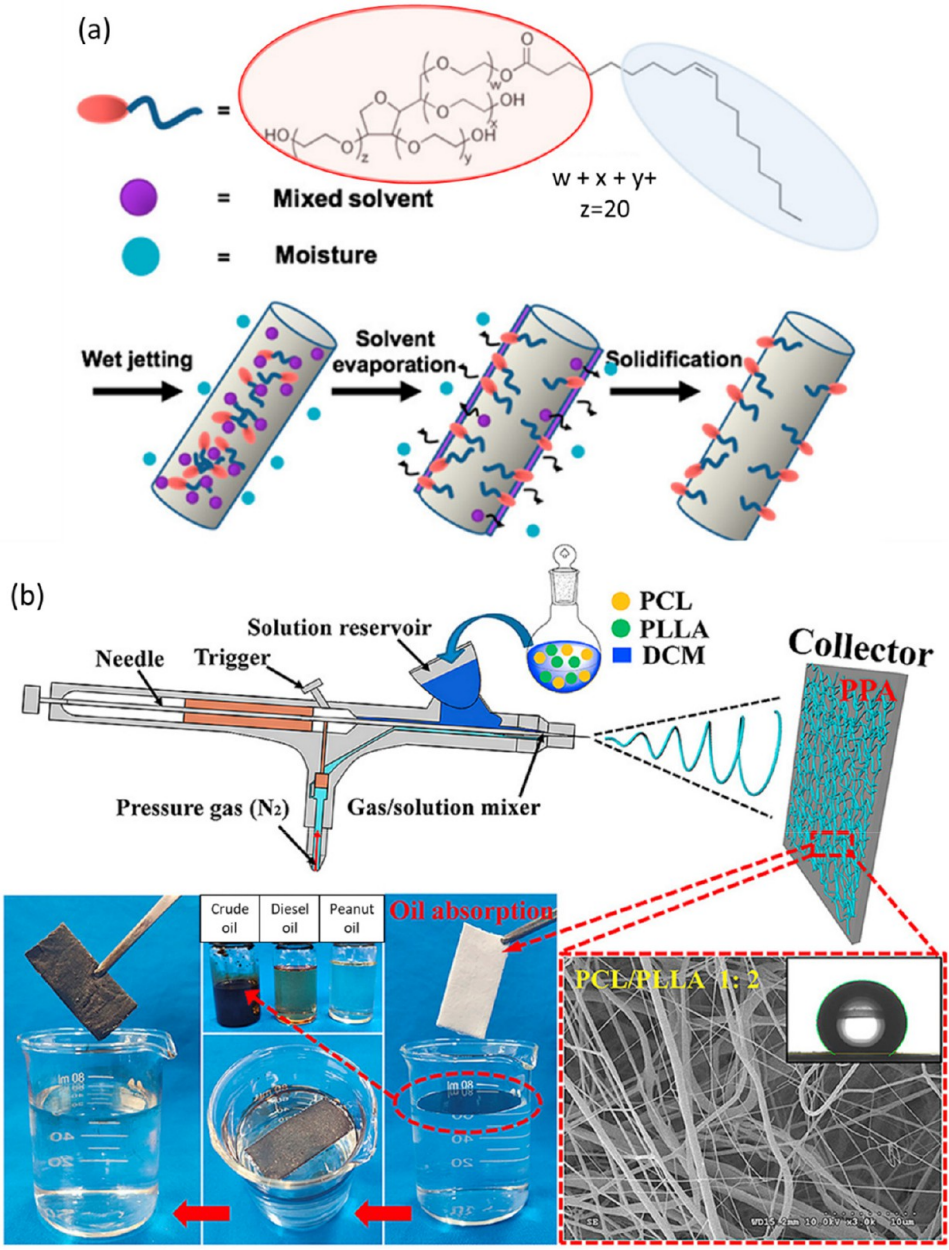
(a) Schematic representing the hydrophilic surface modification of the PCL fiber (cylinder in the figure) with Tween 80. Reproduced with permission from ref (70). Copyright 2017 American Chemical Society. (b) Schematic for the fabrication of a PCL/PLLA composite micro- or nanofibrous membrane through the solution blow spinning technique and its efficacy in oil–water treatment. Adapted with permission from ref (73). Copyright 2020 Elsevier.

Panatdasirisuk et al. prepared membranes from electrospun PCL fibers and Tween 80, a hydrophilic surfactant.^70^ The resulting membranes had high porosity (approximately 88%) and excellent mechanical strength. Upon stretching of the membranes at different strain levels, the pores became anisotropic with an increased aspect ratio. These strained membranes could separate oil–water emulsion droplets as small as 18 nm and maintained a flux of about 70 L/m^2^·h·bar. The membranes exhibited an oil rejection (under gravity) of about 99.0%. Refer to figure 6(a) for schematics.

Jose et al. synthesized TiO_2_-incorporating PCL-based ultrafiltration membranes via the phase inversion technique.^133^ TiO_2_ is particularly known for its ability to impart hydrophilicity, porosity, antibacterial, and antifouling properties. The membranes having 16.5 wt % PCL and 1.5 wt % TiO_2_ exhibited a tensile strength of 4.25 N/mm^2^ and pure water flux of 130 L/m^2^·h at a pressure of 10 bar. Cooper et al. developed a PCL–chitosan-based nanofibrous membrane for antibacterial water filtration.^74^ The resulting membranes were found to reduce *Staphylococcus aureus* bacterial colonization by 50%, along with 100% removal of 300 nm particulates.

Reshmi et al. demonstrated the fabrication of novel superhydrophobic and super oleophilic electrospun nanofibrous membranes from PCL and beeswax.^71^ The designed membranes showed higher sorption capacities for gingelly (25.17 g/g) and sunflower oil (31.05 g/g) than petrol (149.38 g/g), kerosene (20.72 g/g), and diesel (16.95 g/g) compared to the pristine PCL electrospun membrane. Even after fifteen sorption cycles, the electrospun membrane showed a higher sorption capacity. Gravity-driven oil–water separation of these membranes exhibited high flux and a high separation efficiency of 98.1%.

Benhacine et al. studied the possibility of using a PCL membrane for heavy metal removal.^75^ They incorporated PCL with silver-exchange montmorillonite for wastewater remediation and reported sharp declines in nitrates, sulfates, Pb, Zn, and Cd by 15.12%, 45.61%, 41.38%, 53.57%, and 61.11%, respectively.

Nivedita et al. studied the effect of unmodified and modified montmorillonite (MMT) on the properties of PCL-based ultrafiltration membranes to remove Congo red dye from synthetic wastewater.^77^ PCL membranes were subjected to a pressure of 8 bar. The membranes with unmodified MMT showed a pure water flux of about 1500 L/m^2^·h and nearly 35% rejection of Congo red dye. However, the PCL membranes incorporated with modified MMT portrayed a pure water flux of about 400 L/m^2^·h and nearly 45% rejection of Congo red dye. In a different work by the same group, PEG 400 and TiO_2_ nanoparticles were used to tune the PCL membranes’ hydrophilicity and porosity.^134^ The antifouling nature of the prepared membranes was demonstrated via a protein solution (BSA) filtration. The neat water flux (pressure of 8 bar) values for the PCL-PEG and PCL-PEG-TiO_2_ membranes were found to be 129 and 107 L/m^2^·h, respectively. The irreversible fouling ratio for the PCL-PEG-TiO_2_ (∼10%) membrane was smaller than that for the PCL-PEG (∼25%) membrane due to the reduced interaction of BSA with the PCL-PEG-TiO_2_ membrane surface, owing to the presence of hydrophilic TiO_2_ nanoparticles.

Semiromi et al. prepared superhydrophobic and superoleophilic PCL-based membranes via an electrospinning technique for oil–water separation.^72^ They incorporated methanol and silica nanoparticles in optimum proportions, and the resulting membranes were found to exhibit an oil–water separation efficiency of about 98%.

Palacios et al. designed a novel, green, and eco-friendly filtration system based on PCL and cellulose nanofibers (CNF) (obtained from agave bagasse) via an electrospinning technique.^76^ The water quality variables evaluated after filtration with the prepared PCL/CNF membranes showed 100% turbidity removal, 100% conductivity, and heavy metal removal on the order of 75–99% for iron and chromium. Li et al. fabricated composite micro- and nanofibrous membranes for oil adsorption through solution blow spinning using PCL and PLLA.^73^ The prepared micro- and nanofibrous membranes (PPA) demonstrated a higher oil adsorption capacity than the individual raw materials, with values of 24.56, 14.54, and 13.28 g/g to crude oil, peanut oil, and diesel oil, respectively. The oil adsorption capacity could remain about 50% after ten cycles of reuse. Refer to Figure 6(b) for schematics.

### 4.3 Poly(lactic acid) (PLA) Based Membranes

Poly(lactic acid) (PLA) is a polyester, usually derived from fermented maize starch. It represents one of the most commonly used biodegradable polymers. It exists in three isomeric forms, d-form (−), l-form (+), and the racemic mixture (d,l)-forms.^15^ Poly(d,l-lactide) (PDLLA) is amorphous, whereas PLLA and poly(d-lactide) (PDLA) are semicrystalline. PLA degrades via hydrolysis in the presence of moisture. Apart from its renewability and degradability, PLA possesses superior thermal processability, tensile strength, and elastic modulus and is a suitable green substitute for commercially used petroleum-based polymers.^135^

**Figure 7:**
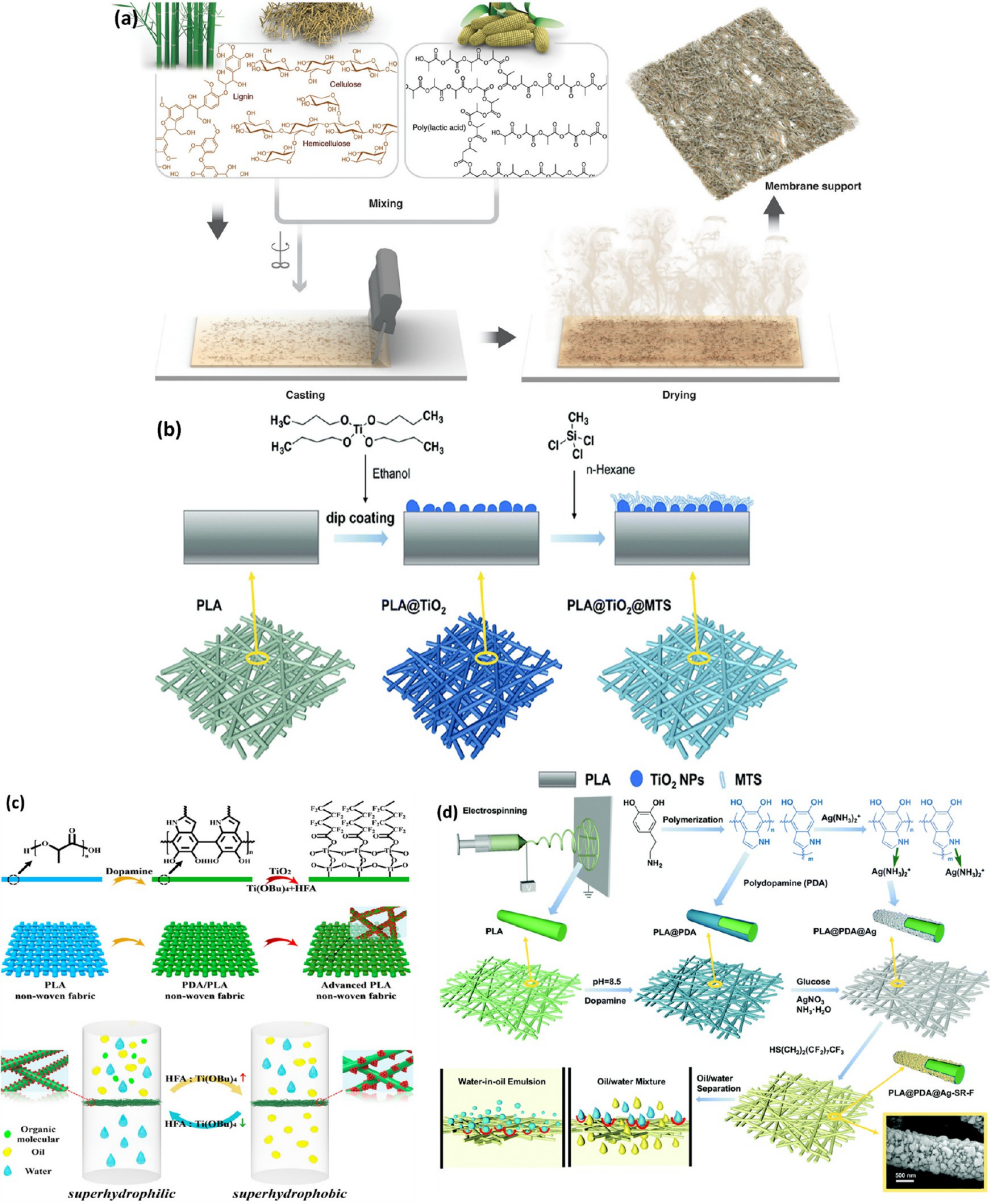
(a) Schematic overview of the PLA/bamboo fiber membrane fabrication. Reproduced with permission from ref (136). Copyright 2019 American Chemical Society. (b) Schematic representation of a PLA@TiO_2_@MTS-functionalized nanofibrous membrane. Reproduced with permission from ref (78). Copyright 2019 Royal Society of Chemistry. (c) Schematic overview of advanced PLA nonwoven fabric for oil–water separation, where PLA was modified with PDA and TiO_2_ nanoparticles. The wettability was tailored with the relative proportion of Ti(OBu)_4_ and HFA, and the obtained fabrics were applied in oil–water separation. Reproduced with permission from ref^140^. Copyright 2018 American Chemical Society. (d) Schematic illustration for the PLA@PDA@Ag-SR-F nanofibrous membrane’s fabrication process and its application in oil–water separation. Reproduced with permission from ref (141). Copyright 2018 Royal Society of Chemistry.

Phuong et al. formulated a bamboo fiber/PLA composite as a sustainable, biodegradable, nonwoven backing material for polymer membranes with a superior flux (pressure of 1 bar) of 1068 ± 32 L/m^2^·h bar and stability in aprotic green solvents like cyrene, P.C., γ-valerolactone, and 2-methyltetrahydrofuran.^136^Figure 7(a) gives a schematic overview of their membrane.

Ahmed et al. reported the fabrication of a PLLA-based nanofibrous membrane using a considerable dosage of polypropylene carbonate (PPC) and PHB. Wastewater containing clay particles and fine sand was filtered using the as-prepared membranes. Interestingly, most of the sand and clay particles were found to be attached to the fibers, thus proving it to be a low-cost filtration approach for wastewater treatment in the agricultural field.^137^

Zhou et al. designed a novel and environmentally friendly superhydrophobic/super oleophilic nanofibrous electrospun membrane with excellent oil–water separation by coating a PLA nanofiber film with TiO_2_ nanoparticles by the sol–gel method and further modified it using methyl trichlorosilane (MTS). The functionalized membrane exhibited excellent superhydrophobicity (water contact angle of 157.4 ± 0.91°), high permeation flux (2297.6 ± 51.6 L m^–2^ h^–1^), and ideal filtration efficiency (98.4 ± 1.0%). The separation was solely gravity driven, and the membranes also achieved rapid and recyclable adsorption of toxic dyes such as methylene blue in an aqueous solution.^78^ Refer to Figure 7(b) for schematics.

Galiano et al. reported the fabrication of flat green sheet PLA via evaporation-induced phase separation (EIPS) followed by nonsolvent-induced phase separation (NIPS). The as-prepared membranes were tested in pervaporation (PV) for the separation of a methanol (MeOH)/methyl *tert*-butyl ether (MTBE) azeotropic mixture by varying the feed temperature and the vacuum degree. The PLA dense membrane produced with an evaporation time of 7 min was successfully tested in PV, exhibiting a selective permeation toward MeOH with the highest selectivity value of more than 75.^81^

A recent study by Su et al. made use of a stereocomplex PLA membrane via the nonsolvent-induced phase separation technique. The membrane was super hydrophobic in nature and exhibited a water contact angle of 152°. The membrane was found to absorb oil 4–5× its own weight and could absorb cyclohexane within 10 s of dipping into the oil baths.^138^

Krasian et al. made composite mats of PLA and a hybrid of 2D materials, i.e., MoS_2_ and WS_2_, for oil adsorption and oil–water separation. The mats exhibited an increased oil adsorption capacity of about 190% when compared to neat PLA mats. The composite mats were also found to behave as oil–water separators by functioning as oil adsorbents for floating oil on water surfaces and acting as separation membranes in a simple gravity-driven filtration system. Nearly 70% flux recovery is obtained for the effective separation of a surfactant-stabilized oil–water emulsion.^79^

Gao et al. fabricated a biomimetic, robust, and superhydrophobic poly(l-lactic acid) (PLLA) based membrane with an urchin-like hierarchical surface having superior antiwetting characteristics. Using a simple phase inversion method led to forming a bacteria-resistant membrane surface and self-cleaning characteristics without incorporating any fluorine components.^82^ Among other notable works, Moriya et al. developed hollow fiber ultrafiltration membranes from PLLA using PLLA–dimethyl sulfoxide (DMSO) solutions with relatively high water permeability (324 ± 46 LMH) (pressure range of 0.05–0.1 MPa) along with a bovine serum albumin rejection of about 80%.^83^

Takahashi et al. developed microfiltration membranes from a polymeric blend of PLLA and PCL. A blend ratio (PCL/PLLA) of 4:1 was found to retain yeast cells without exfoliating the membrane.^20^ Similarly, bacterial rejection achieved using PLA-based membranes was investigated by Jalvo et al. With the aid of electrospinning, they synthesized core–shell nanofibers from PLA and PAN (polyacrylonitrile)/cellulose nanocrystals. Within the range of 5–15 wt% loading of the nanocrystal, the best mechanical property enhancements were recorded. The coaxial membranes rejected 85% of bacterial cells and 99% of fungal spores.^139^

Peng et al. developed a simple strategy to obtain biodegradable PLA nonwoven fabric with controllable wettability for efficient water purification from oil–water mixtures. The resultant material is characterized by a high absorption capacity and selectivity, a photodegradation property, and biodegradability. The superwettability of PLA made the advanced nonwoven fabric a promising candidate for oil–water separation. The controllable wettability between superhydrophobicity and super hydrophilicity allowed the utilization of the PLA nonwoven fabric to switch selective oil–water separation.^140^ Refer to Figure 7(c) for schematics.

Hassani et al. blended PDLLA with PESU to prepare asymmetric membranes via the phase inversion method. The mere addition of poly(ethylene glycol) (PEG) as an additive improved the membrane hydrophilicity, flux rates, and biodegradability compared to PDLLA alone. The resulting membrane additionally showcased a 99% retention of a *L. plantarum* cell suspension.^84^

Yuan et al. fabricated a hierarchal electrospun nanofibrous PLA membrane coated with polydopamine (PDA) combined with silver nanoparticles and fluorinated thiol hydrophobic functionalization (PLA@PDA@Ag-SR-F).^141^ The resulting membranes exhibited high permeation fluxes (2664 L/m^2^·h) for a wide variety of oils and a desirable separation performance (separation efficiency of >95%) for both different oil–water mixtures and surfactant-stabilized water-in-oil emulsions. Apart from being highly recyclable, these membranes’ antibacterial activity against *Escherichia coli* and *S. aureus* reached nearly 100%. Refer to Figure 7(d) for schematics.

Liu et al. designed a highly porous electrospun PLA membrane wherein the porosity was induced by humidity.^142^ The fabricated membrane was meant for potential oil–water separation applications. An increase in the pore size led to an increase in the oil permeation flux. The resulting membranes showcased a separation efficiency of about 99.98% for three oil types, i.e., *n*-hexane, olive oil, and lubricant oil. Chen et al. prepared a novel PLA/PEI (polyethyleneimine) membrane via a dip-coating technique for simultaneous and efficient dye and oil–water separation.^143^ The membranes showed a separation efficiency of higher than 99.7% for three actual oils (methylbenzene, hexamethylene, and soyabean oil). Moreover, the obtained membranes could realize the high-efficiency and simultaneous removal of methyl orange dye (MO) (removal efficiency >99.8%) and oil droplets (oil rejection >99.7%) from mixed wastewater under a permeate flux of up to 8285 L/m^2^·h solely driven by gravity.

Xing et al. designed a 3D printed lotus leaf-inspired superhydrophobic PLA membrane for oil–water separation.^80^ The membrane exhibited an excellent oil–water separation efficiency of over 99% while retaining a high flux of 60000 L/m^2^·h (driven via gravity). Gu et al. fabricated a PLA/SiO_2_/PS (polystyrene) hybrid nonwoven fabric with a hierarchical porous structure with a high oil absorption capacity for the selective separation of oil–water mixtures.^144^ It quickly absorbs *n*-hexane and tetrachloromethane floating on the water within 3 s. The material was found to be recyclable even after ten cycles of use.

Yu et al. synthesized robust and porous PLA membranes with improved mechanical and thermal stability via incorporating polysulfone-graft-PLA (PSF-g-PLA) copolymer.^85^ These modified PLA membranes exhibited a pure water flux of 54 L/m^2^·h (pressure of 0.1 MPa), 95% rejection to BSA, and 65% and 18% clearance of urea and lysozyme, respectively.

Ji et al. utilized polyvinylpyrrolidone and glutaraldehyde as cross-linkers to prepare stable PLA/Ag/AgCl composite photocatalytic membranes via electron beam irradiation.^145^ The membranes exhibited favorable antibacterial activity against *E. coli* and *S. aureus*, with a bacterial removal rate of 77%. Zhang et al. developed a controllable approach for achieving super hydrophilicity of biodegradable electrospun stereocomplex polylactide (sc-PLA) membranes via titanium carboxylate coordination bonding of gallic acid (GA) and tetrabutyl titanate (Ti(OBu)_4_) in an aqueous solution, i.e., GA-modified TiO_2_ (GA-TiO_2_) coating.^146^ The membranes showed an oil–water separation efficiency of 99.6%, along with a permeation flux of 4200 L/m^2^·h for the *n*-hexane-in-water emulsion. For methylene blue aqueous solution, the resulting membranes showed a flux of 636 L/m^2^·h driven by gravity.

Aseri et al. investigated the morphology and separation performances of PLA-modified polyvinylidene fluoride (PVDF) hollow fiber membranes.^103^ Results indicated that incorporating a small quantity of PLA could improve the membrane water flux from ∼30 to 376.7 L/m^2^·h·bar at a pressure of 1 bar without compromising humic acid (HA) rejection (95–97%). Improved surface hydrophilicity (indicated by a lower water contact angle) led to a higher flux recovery rate than the pure PVDF membrane, revealing the improved antifouling resistance against bovine serum albumin.

Zhu et al. designed a superhydrophobic and super oleophilic PLA nonwoven fabric with stereo complex crystals for oil–water separation applications.^147^ The resultant material demonstrated a separation efficiency of 97% and retained its high flux and separation efficiency even after ten usage cycles. Yin et al. designed highly porous 3D PLLA nanosheets, fibrous nanosheets, and nanofibrous networks via gradual precipitation for oil–water separation application.^148^ Owing to hydrophobicity, high porosity, and excellent capillary effect, the porous materials’ oil absorption ratio was found to reach more than 2900%, which was significantly higher than the absorption capacity of materials obtained via the traditional thermal-induced phase separation method.

Khalil et al. designed ultrafiltration membranes using PLA for the removal of organic substances from wastewater. The membranes demonstrated an 89% flux retention ratio against model protein BSA while being able to demonstrate 82% chemical oxygen demand.^149^ Nassar et al. fabricated PLA ultrafiltration membranes using NIPS on a nonwoven polyester support. The membranes were able to remove 96% of NH_4_^+^–N and up to 52% of PO_4_^3—^P.^150^ Ampawan et al. fabricated a unique membrane by blending carboxylated cellulose PLA and poly(butylene adipate-*co*-terepthalate) using NIPS. This membrane achieved a flux of 1214 L/m^2^·h with 97.2% MB rejection.^151^ Using poly(l-lactic acid) and modified natural halloysite nanotubes, Wang et al. fabricated hybrid membranes for the rejection of proteins. With a model BSA protein foulant, although the rejection of BSA decreased to 82.7% from 94.3% with just 1% nanotube loading, the antifouling resistance increased.^152^ Thus ,nanomaterials and nanotechnology in general can greatly impact the performance of the systems and revolutionize water treatment operations.^153^

### 4.4 Poly(hydroxyalkonate) (PHA) Based Membranes

PHAs represent a family of biopolyesters formed by several microorganisms.^154^ Highly crystalline PHB is a well-characterized homopolymer, but it is inherently brittle and has a narrow processing window.^155^ To overcome the aforementioned limitations, several copolymers of PHB such as PHBV are typically produced. These newly synthesized PHAs showed pronounced biocompatibility and biodegradability and had better mechanical properties than pristine PHB.^156^ It has been observed in several research works over the years that most wastewater treatment is based on aerobic and anaerobic microbial decontamination. In this regard, nanofibrous filtration membranes can act as a potential solution for wastewater treatment.

Marova et al. reported the fabrication of a biodegradable PHA nanofibrous membrane using electrospinning and spin-coating techniques. Following this, they introduced certain functional groups into the PHA backbone. The resulting membrane showed filtration efficacies of about 81.9%, 63.7%, 32.1%, and 74.2% for the bacterial strains *B. subtilis*, *M. luteus*, *E. coli*, and *S*. *cerevisiae*, respectively. Promising results were obtained for Gram-positive and Gram-negative strains, which hinted at the membrane’s efficiency for microorganism removal.^86^

Tomietto et al. synthesized a microfiltration membrane based on PHBV via an evaporation-induced phase separation technique (EIPS). The membranes were modified by using different molecular weights, PEG concentrations, and PVP to tune the membrane performance and characteristics. Results indicated that the membrane with PEG8000 showed superior performance, with pure water permeabilities over 200 L/m^2^·h·bar (pressureof = 2 bar) associated with a bacteria rejection of 99.95%.^87^

Fan et al. prepared and characterized electrospun fibrous membranes based on PHB with superior antimicrobial properties. The biocidal properties were incorporated after exposure to a chlorine bleach. The resulting membranes were found to inactivate 92.10% *S. aureus* and 85.04% *E. coli* O157:H7 within 30 min of contact time.^88^

### 4.5 Poly(vinyl alcohol) (PVA) Based Membranes

PVA is a nontoxic, biocompatible, biodegradable, and water-soluble synthetic polymer that is easily processable. PVA is not synthesized through the direct polymerization of its monomer, vinyl alcohol. Poly(vinyl acetate) is prepared first and then converted to PVA, since the monomer unit of PVA is not thermodynamically stable. It potentially forms miscible homogeneous systems and exhibits exceptional chemical resistance, film-forming ability, and hydrophilicity.^157^ It is highly applicable for the formation of pressure-driven membranes for desalination purposes. The highly polar nature of PVA is instrumental in minimizing fouling in such applications. Because of its superoleophobic properties, PVA serves a superior candidate in wastewater remediation, product recovery, and separation of organic compounds from one another or water. Owing to their innate ability to swell in aqueous solutions, PVA membranes have to be modified. Modifications such as cross-linking, blending, and incorporating inorganic fillers have been widely accepted to improve PVA’s water insolubility and swelling characteristics.^158−160^

Gonzalez-Ortiz et al. developed a novel and porous PVA membrane via Pickering emulsion templating using hexagonal boron nitride nanosheets (h-BNNs) as a stabilizer.^89^ The resulting membranes displayed a pore size of nearly 1 μm with a water permeability over 2000 L/m^2^·h·bar and a rejection efficiency of ∼100% for different latex NPs.

Yoon et al. fabricated a high flux thin film nanocomposite membrane (TFNC) based on polyacrylonitrile (PAN) electrospun scaffolds and cross-linked PVA coating.^90^ The resulting ultrafiltration (UF) membranes exhibited a very high flux (nearly 12× higher than conventional UF PAN membranes). They also exhibited a rejection ratio of >99.5% for oil–water mixture separation.

**Figure 8:**
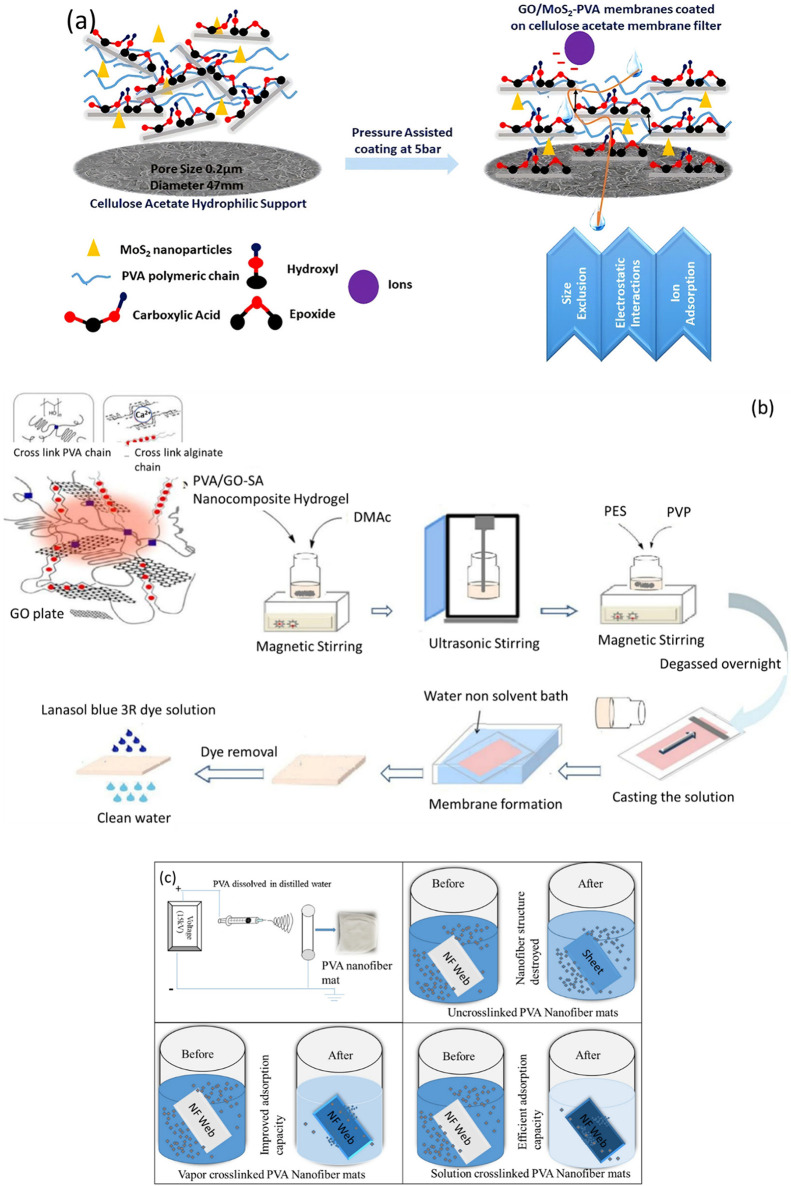
(a) Schematics for the fabrication of GO/MoS_2_–PVA composite membranes. Reproduced with permission fromref (93). Copyright 2020 Elsevier. (b) Schematics for the synthesis of asymmetric microporous polyethersulfone (PES)/polyvinyl alcohol–graphene oxide–sodium alginate (PVAGO-NaAlg) nanocomposite hydrogel (HG) blended nanofiltration membranes. Adapted with permission from ref (95). Copyright 2020 Elsevier. (c) Schematic for the mechanism of heavy metal removal. Reproduced with permission from ref (99). Copyright 2019 Elsevier.

To treat landfill leachate water, Yadav et al. developed GO/MoS_2_-PVA composite membranes for NaCl rejection, toxic heavy metal, and radioactive element removal.^93^ These composite membranes had an 89% rejection rate to NaCl and a water flux of 3.96 L/m^2^·h at an operating pressure of 5 bar. Rejection rates of nearly 86.5–99.8% were observed for multivalent metal ions in landfill leachate for a particular membrane composition. Refer to Figure 8(a) for schematics.

Amiri et al. synthesized novel asymmetric microporous polyethersulfone (PES)/polyvinyl alcohol–graphene oxide–sodium alginate (PVA-GO-NaAlg) nanocomposite hydrogel (HG) blended nanofiltration membranes for water remediation purposes.^95^ The hydrogel blended membranes showed a Lanasol blue 3R dye rejection of more than 83%. Membranes with 1 wt % PVP (polyvinyl pyrrolidone) and 1 wt % HG showed a pure water flux of 115.7 L/m^2^·h (pressure of 3 bar) and a BSA rejection of nearly 99%. Refer to Figure 8(b) for schematics.

Ullah et al. designed a novel solution cross-linking technique to prepare PVA nanofibrous mats, which targeted enhanced adsorption of Cu^2+^ and Pb^2+^ metal ions.^99^ The as-prepared mats demonstrated adsorption capacities of 58.3 mg/g for Cu^2+^ ions and 161.7 mg/g for Pb^2+^ ions. Refer to Figure 8(c) for the schematics.

Zhang et al. developed a cost-effective electrospun PVA/lignin composite nanofibrous membrane as a high-performance adsorbent for adsorbing the cationic dye safranine T for water purification.^161^ The resulting adsorbent exhibited excellent desorption behavior in a sodium hydroxide solution, with an optimal desorption time of 4 h. It could be successfully recycled with a stable adsorption performance for five successful runs.

Ghaffar et al. reported a versatile porous PVA/GO nanofibrous membrane prepared via electrospinning for remediating oily water.^91^ The membranes achieved a separation efficiency of >99% for surfactant-free and surfactant-stabilized oily water emulsions. They also demonstrated a water flux of about 45 L/m^2^·h oil–water emulsions and that of 30 L/m^2^·h for surfactant-stabilized oil–water emulsions entirely under gravitational force due to the presence of GO.

Ebrahimi et al. fabricated composite nanofibers based on PVA and sodium alginate to remove cadmium ions from aqueous solutions.^162^ Although the maximum amount of equilibrium adsorption under optimum experimental conditions was 67.05 mg/g, the maximum adsorption capacity through the Langmuir model was found to be equal to 93.163 mg/g adsorbent.

Sabarish et al. fabricated an efficient and biodegradable PVA/carboxymethyl cellulose (CMC) and ZSM-5 zeolite membrane to remove MB dye.^96^ The membranes demonstrated a high dye removal efficiency (97%) and high adsorption capacity (7.83) for the 5 wt % zeolite-loaded sample for an initial dye concentration of 10 ppm and a contact time of 10 h at 30 °C.

Cheng et al. reported a highly perm-selective networked membrane composed of PVA/GA (glutaraldehyde)/CS (chitosan)–Ag^+^ for isopropanol dehydration.^100^ The membrane with a Ag^+^ content of 1.17 × 10^–2^ mol, and a very high flux of 1.97 kg/m^2^·h, was accompanied by a separation factor up to 89991 for the pervaporation dehydration of an isopropanol solution with the concentration of 90 wt % under a temperature of 30 °C.

Zhang et al. prepared PVA/SiO_2_-modified stainless steel mesh to be used as an underwater superoleophobic membrane for efficient oil–water separation.^92^ The mesh’s maximum separation efficiencies for hexane, cyclohexane, diesel, soybean oil, lubricating oil, and silicone oil were 99.4%, 99.3%, 99.6%, 99.0%, 98.6%, and 98.0%, respectively.

Fan et al. designed thermostable and hydrostable zeolitic imidazolate framework-8@PVA nanofibrous membranes with good mechanical properties and adsorption properties via an electrospinning technique.^97^ The membranes achieved a separation efficiency of nearly 95% for Congo red dye.

Barona et al. demonstrated the fabrication of a novel thin film nanocomposite (TFN) nanofiltration membrane by incorporating aluminosilicate single-walled nanotubes (SWNTs) within the PVA matrix.^94^ Owing to the inclusion of aluminosilicate SWNTs, a higher permeate water flux was achieved while still sustaining high rejection of divalent ions (97%) and monovalent ions (59%).

Lin et al. thoroughly investigated nanosilica/PVA composite membranes to deliver an improved caprolactam pervaporation (PV) dehydration process.^163^ The evaluated results demonstrated a maximum flux of 3.8 kg/m^2^·h and an acceptable separation factor of 150.

Shirazi et al. synthesized and characterized nanocomposite membranes based on carbon nanotubes (CNTs) and PVA.^164^ Results indicated that incorporating CNTs significantly improved water selectivity due to polymer chain rigidity; the water selectivities for the pristine PVA and 2 wt % CNT loading nanocomposite membranes were evaluated as 119 and 1794, respectively. Zendehdel et al. prepared a novel semi-IPN composite hydrogel from PVA, copolymer acrylic acid, and acrylamide to remove MB dye from wastewater.^98^ The maximum dye adsorption concentration for the hydrogel composites was evaluated to be 95%, and no dye desorption of MB/polymer solutions was observed.

## 5 End-of-Life Considerations of Biodegradable Membranes

From the above examples, it is clear that the polymers above possess a high potential for water remediation applications. However, it is also important to consider these polymers’ fate after they are no longer used for water treatment. A common misconception is that all biodegradable plastics (including those of cellulose and chitin) can be treated using organic waste management (composting).^23^ Compostability and biodegradability are used interchangeably, and end-users may segregate them inappropriately, leading to waste generation instead of closing the recycling loop. The sole use of the term biodegradable gives a direct assumption that it will degrade irrespective of environmental conditions, but this is far from the truth. Biodegradation is defined as the breakdown of the polymeric material by microbes, such as bacteria and fungi. Under anaerobic conditions, the polymer material is converted to biomass, carbon dioxide, water, and methane, while in aerobic conditions carbon dioxide, biomass, and water are formed due to biodegradation.^23,165^ The biodegradation rate of any polymeric material is critically dependent on environmental conditions like temperature, nutrient composition, pH, oxygen supply, microbial consortia, and activity.^15,23^ On the contrary, according to ASTM D6400, EN13432, and ISO 17088, compostable polymers must be biodegradable and have no residual ecotoxicity associated with them.^166^

**Figure 9:**
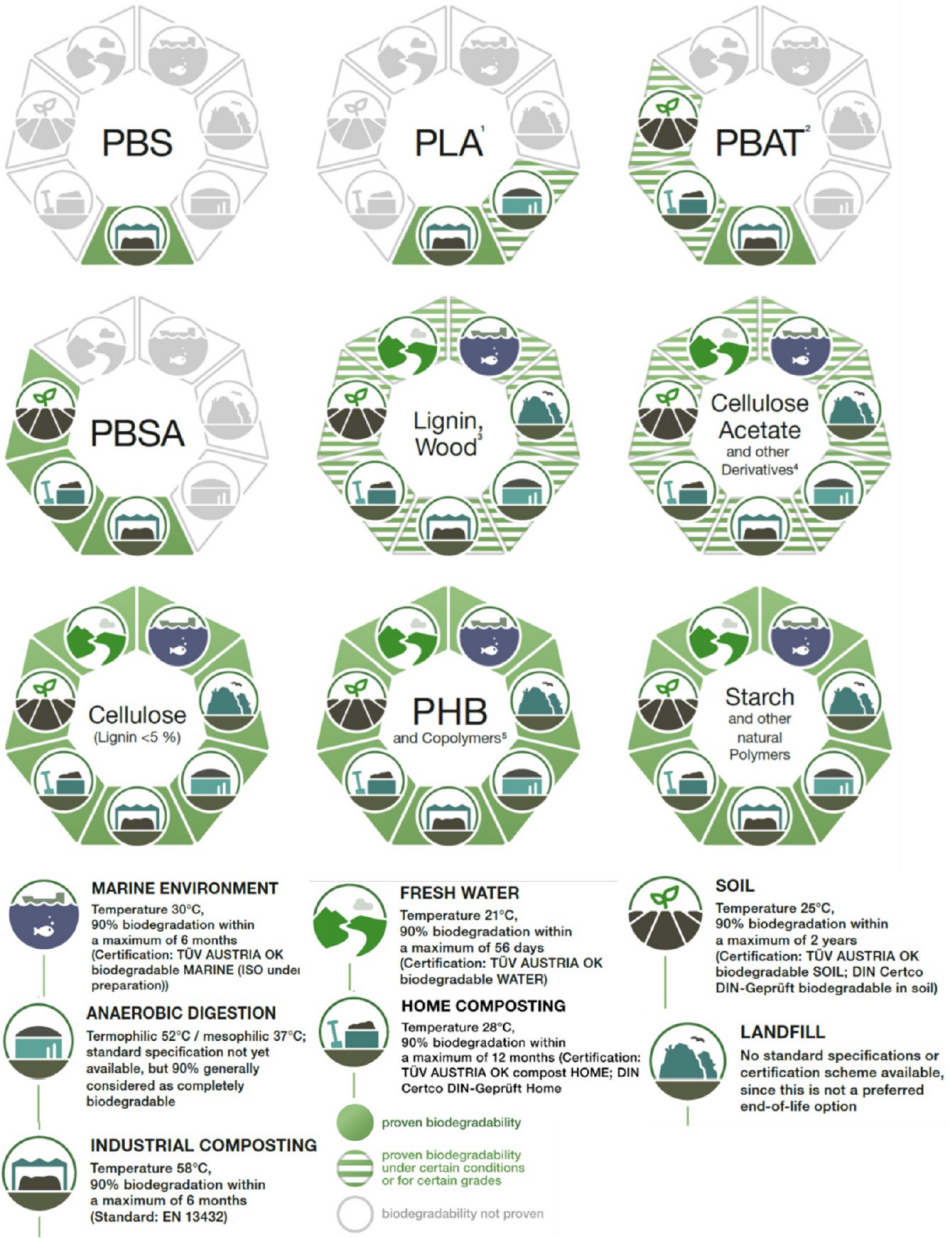
Biodegradability of common biodegradable polymers in different test environments. Adapted from an open-to-use study by nova-Institute under Renewable Carbon Publications.^173^ Footnote as per nova-Institute: ^1^PLA likely biodegrades in at temperature above 52 °C (thermophilic anaerobic digestion). ^2^Biodegradability of PBAT in soil and home composting was only demonstrated for certain polymer grades. ^3^For high lignin, complete biodegradation is not easily measurable with standard biodegradation tests but does take place (slowly). Humus is a byproduct instead of CO_2_ after biodegrading lignin-rich materials. ^4^Only certain grades of CA are proven to biodegrade in all environments. ^5^Classification includes copolymers of PHB such as P3HB, P4HB, P3HB4HB, P3HB3HV, P3HB3HV4HV, P3HB3Hx, P3HB3HO, and P3HB3HD.

A recent perspective by Samantaray et al. highlights the differences between biodegradability and compostability, test methods to evaluate compostability and biodegradation, and methods to tailor the compostability of synthetic biopolyesters by copolymerization and blending in detail.^23^ A mini-review by Haider et al. discusses the biodegradation of different biodegradable polymers by microbes and highlights the key fact that biodegradable materials show different degradation in different environments. Further biodegradation in artificial conditions lacks transferability when used in real-world applications.^167^ For example, PLA, which is categorized as biodegradable, is only decomposable to constituent biomass in industrial composting conditions and can take decades to degrade in home composting, soil, and landfill conditions (<37 °C).^168,169^ For PVA, which is stable in sludge, enzymes (e.g., secondary alcohol oxidase from the *Pseudomonas* strain), bacteria like *Brevibacterium*, *Pseudomonas*, *Alcaligenes*, and *Bacillus megaterium*, fungi like *Aspergillus*, *Fusarium*, and *Phanerochaete chrysosporium* are effective in the biodegradation and assimilation of PVA.^170,171^ As per Rodica et al., at least 55 species of microbes like bacteria, fungi, yeast, and mold can participate in the biodegradation of PVA.^170^Figure 9 shows the biodegradation of common biopolymers in various environmental conditions. It should be noted here that the conditions for biodegradation shown here are essentially for the polymers in their pristine form. Blending, copolymerization, and modifications alter the biodegradation characteristics of the modified polymer system.^23^ After the water treatment process, waste contaminants, such as recalcitrant dyes, pharmaceutical wastes, pathogenic microbes, and ions, will dominate the disposal considerations. Life cycle assessments of such scenarios must be done for a sustainable closed-loop economy.

The European Bioplastics webpage houses free-to-use information regarding basic facts and figures as well as background papers and market data on bioplastics. Enzyme degradation, certification schemes, composting conditions, mechanical and chemical recycling, energy recovery, landfilling, and anaerobic digestion are also covered on the webpage. Since the discussion of these considerations goes beyond this article’s scope, readers are encouraged to visit the web page for more information.^172^

## 6 Conclusions and Outlook

Membrane technology is highly effective for meeting stringent water quality standards; however, there is a concern over the fate of these membranes after use, as they are nonbiodegradable and their disposal can have a negative impact on the environment. Biodegradable bioplastics can potentially address this issue since they degrade over time under controlled environmental conditions and have low carbon emissions. However, to replace existing membrane materials, biodegradable materials must surpass their limitations and exhibit properties comparable to those of conventional membrane plastics. These limitations include mechanical brittleness and poor water stability.

In assessing the feasibility of biodegradable bioplastics for water remediation, it was observed that while they possess similar or superior characteristics compared to conventional membrane materials, they have poor mechanical, thermal, and water stabilities. Unique approaches such as copolymerization and cross-linking techniques can be used to tailor their mechanical properties while retaining water stability. Nanoparticles and polymer blending can also be used to exploit existing materials alongside bioplastics. While cellulosic and chitosan derivatives have been used extensively for water remediation applications, recent years have seen the realization of other biodegradable materials such as PBS, PCL, PLA, PHA, and PVA for membrane applications in wastewater, heavy metal removal, and oil–water separation. The end-of-life of these polymers is also important to consider.

Future research directions may include improvising water permeation, improving the shelf life, and tailoring foul resistance performance. For instance, stable mixed-matrix membranes can be formed by blending biodegradable materials with water- and temperature-stable metal–organic frameworks and covalent organic frameworks. Natural clay and nanowiskers as well as 2D layered materials can also be modified to improve separation characteristics. Polymer blends and interpenetrating cross-linked polymer networks exploring conventional polymer blends with a biodegradable polymer component can also be a way forward. Finally, the evaluation of the biodegradability of polymer membranes after their intended use should be explored further to reduce plastic waste pollution. Designing and developing biodegradable bioplastics will accelerate the reduction of plastic waste and lead to a cleaner and more sustainable ecosystem.
